# Enterohaemorrhagic *Escherichia coli* AdhE spirosome length correlates with enzymatic directionality and is perturbed by salicylidene acylhydrazides

**DOI:** 10.1098/rsob.250041

**Published:** 2025-06-18

**Authors:** Ester Serrano, Tianxiao Zhao, David R. Mark, Mostafa Soroor, Iris Floria, Nicholas J. Terrill, Nikil Kapur, Arwen I. I. Tyler, Mathew H. Horrocks, Andrew J. Roe, Olwyn Byron

**Affiliations:** ^1^ School of Infection and Immunity, University of Glasgow, Glasgow G12 8TA, UK; ^2^ EaStCHEM School of Chemistry, University of Edinburgh, Edinburgh EH9 3FJ, UK; ^3^ IRR Chemistry Hub, Institute for Regeneration and Repair, University of Edinburgh, Edinburgh EH16 4UU, UK; ^4^ EPSRC CDT in Fluid Dynamics, School of Computing, University of Leeds, Leeds LS2 9JT, UK; ^5^ Diamond Light Source, Harwell Science and Innovation Campus, Didcot OX11 0DE, UK; ^6^ School of Mechanical Engineering, University of Leeds, Leeds LS2 9JT, UK; ^7^ School of Food Science and Nutrition, University of Leeds, Leeds LS2 9JT, UK

**Keywords:** AdhE, analytical ultracentrifugation, small-angle X-ray scattering, salicylidene acylhydrazide, spirosome, total internal reflection fluorescence microscopy

## Abstract

Enterohaemorrhagic *Escherichia coli* causes sporadic, and sometimes large-scale, food poisoning outbreaks, for which antibiotic treatment in humans is contraindicated. As an alternative form of therapy, previous studies developed the family of salicylidene acylhydrazide (SA) anti-virulence compounds. One target of the SA compounds is AdhE, an enzyme that converts acetyl-CoA to ethanol and vice versa. AdhE oligomerizes, forming helicoidal filaments, heterogeneous in length, called spirosomes. We show it is possible to only partially fractionate AdhE spirosomes because *in vitro* they oligomerize in the absence of stimuli, and that spirosome formation is necessary to regulate the direction of AdhE enzymatic reactions. We also show that the SA compound ME0054 binds and perturbs AdhE spirosomes, enhancing the conversion of ethanol to acetyl-CoA. This mechanistic understanding of how ME0054 impacts AdhE function will help in the development of SA compounds as novel anti-virulence inhibitors.

**Figure:**
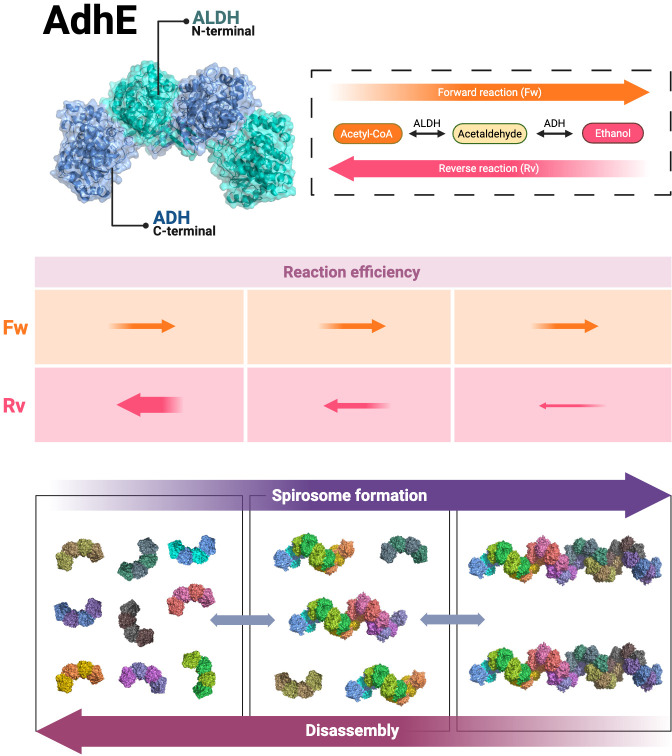

## 1. Introduction

Combatting bacterial infections has become progressively challenging owing to the rising prevalence of antibiotic-resistant strains and a decline in the discovery and development of novel, effective antibacterial agents [1]. Currently, most antibiotics operate by inhibiting enzymes crucial for pathogen survival, e.g. β-lactams and aminoglycosides target bacterial cell wall biosynthesis and translation, respectively [2]. As these processes are essential for growth, the selective pressure imposed by antibiotics is strong, increasing the likelihood of antibiotic resistance [3]. The identification of novel targets not essential for survival is therefore an active area of research [4].

The so-called ‘anti-virulence’ (AV) approach specifically targets virulence factors used by bacterial pathogens to facilitate infection [5]. AV compounds designed or screened to target important virulence traits such as quorum sensing, adhesins and secretion systems have been tested; however, most are at an early stage of development with only a small number reaching clinical trials [6]. AV approaches are particularly advantageous when the use of traditional antibiotics is not appropriate. For example, the clinical symptoms associated with enterohaemorrhagic *Escherichia coli* (EHEC) infections can increase in severity following administration of certain antibiotics [7] as the result of activation of the bacterial SOS response and subsequent release of Shiga toxin [8]. By instead targeting virulence, these negative side effects may be mitigated.

One of the most extensively studied groups of AV compounds is the salicylidene acylhydrazides (SA), a class of inhibitors able to decrease expression of a *yopE*-luciferase transcriptional fusion in *Yersinia pseudotuberculosis*. YopE is a secreted effector protein; therefore, a decrease in luciferase activity from the promoter was correlated with inhibition of the type three secretion system (T3SS) [9], itself a critically important virulence factor used by many Gram-negative pathogens to subvert host cell function and facilitate infection. SA compounds can inhibit the T3SS of numerous pathogens including *Chlamydia trachomatis*, *Salmonella* Typhimurium, *Shigella flexneri* and EHEC [4].

The mode of action of SA compounds has been explored in several studies [4] including our previous work identifying 16 putative SA compound protein targets in EHEC [10]. We systematically deleted the genes encoding the putative target proteins to try to deduce which contributed to the decreased T3SS expression phenotype. These included the gene encoding AdhE, a bifunctional aldehyde–alcohol dehydrogenase that catalyses the conversion of acetyl-CoA to ethanol and vice versa, at biologically relevant temperatures and timescales and is thus a truly bidirectional enzyme [11–14]. The contribution of AdhE to EHEC pathogenesis has been demonstrated in two *in vivo* systems: a rabbit intestinal model and a zebrafish infection model, in both of which the *adhE* deletion mutant was much less infectious than the wild-type strain [11,15]. Deletion of *adhE* results in a 20% increase in extracellular acetate [11] and robust suppression of T3SS expression. These phenotypes are notable as they are similar to those observed when the wild-type strain was exposed to SA compounds [11]. However, how the SA compounds cause this phenotype was unresolved and has spurred us to better understand the structural basis for AdhE function. Moreover, to date, no direct evidence exists to demonstrate that the SA compounds directly bind to AdhE itself.

AdhE oligomerizes *in vivo* and *in vitro* [14], forming long filaments, heterogeneous in length, called spirosomes [16]. The cryo-electron microscopy (cryo-EM) structure of the *E. coli* spirosome has been reported and mutations perturbing its helical structure revealed that spirosome formation is critical for AdhE activity [12]. However, why and how AdhE forms spirosomes heterogeneous in length was still a mystery. We envisioned two hypotheses to explain why AdhE forms spirosomes with different lengths: (a) longer spirosomes are enzymatically more efficient or (b) spirosome length drives enzymatic reaction direction. To test these hypotheses, we fractionated AdhE spirosomes by length; the oligomeric species present in each fraction were analysed by small-angle X-ray scattering (SAXS) and analytical ultracentrifugation (AUC). The efficiencies of fractions containing a main population of long or short spirosomes were compared in the forward and reverse directions of the enzymatic reaction. To understand how AdhE forms spirosomes heterogeneous in length, the dynamics of spirosome formation were observed *in vitro* using total internal reflection fluorescence microscopy (TIRFM). To better understand how the SA compounds impact AdhE functionality, the effect of the SA compound ME0054 [10,17] on spirosome structure and enzymatic efficiency was tested. We show here that ME0054 binds to and perturbs AdhE spirosomes at biologically relevant temperatures and timescales, thereby enhancing the conversion of ethanol to acetyl-CoA. This mechanistic understanding of how ME0054 alters AdhE function will help in the refinement and development of this class of compounds as novel anti-virulence drugs.

## 2. Material and methods

### 2.1. Protein purification and size exclusion chromatography

The *adhE* gene from *Escherichia coli* K12 was cloned into a pET28a vector for expression with an N-terminal 6-His tag. Plasmid expressing recombinant 6-His-AdhE protein was transformed into BL21(DE3) competent *E. coli* cells. A single colony was used to inoculate an overnight culture at 37°C with shaking at 200 rpm prior to back-diluting into 4 l fresh LB supplemented with kanamycin (50 µg ml^−1^) and cultured until OD_600 nm_ = 0.8 was reached. AdhE overexpression was induced by the addition of IPTG to a final concentration of 0.5 mM and cultures were grown overnight at 18°C. Cells were harvested by centrifugation for 10 min at 5000 rpm and the supernatant removed. The cell pellet was resuspended in buffer A (50 mM tris–HCl pH 8.0, 500 mM NaCl, 5% (v/v) glycerol) containing 5 mM imidazole, lysozyme, EDTA-free protease inhibitor cocktail (Promega) and DNase. Cells were lysed by sonication on ice (1 s on, 1 s off for 6 min) and then centrifuged for 50 min at 4°C at 18 000 rpm. After centrifugation, the 6-His-AdhE protein was found in the soluble fraction. The supernatant was removed and filtered through a 0.22 µm filter and loaded onto a HisTrap High Performance column (GE Healthcare) equilibrated with buffer A containing 5 mM imidazole. The column was washed with a linear gradient (5–75 mM) of imidazole in buffer A and 6-His-AdhE was then eluted in 50 ml buffer A with 300 mM imidazole. The 50 ml containing 6-His-AdhE was concentrated to a final volume of 10.5 ml using a Vivaspin 20 centrifugal concentrator (Sartorius) with a molecular weight cut-off of 30 kDa and then dialysed against buffer A. A single aliquot (0.5 ml) of unfractionated 6-His-AdhE in buffer A was stored at −20°C for subsequent investigation using TIRFM.

For protein fractionation by size exclusion chromatography (SEC), the remaining 10 ml of concentrated 6-His-AdhE in buffer A was loaded onto a HiLoad 26/600 Superdex 200 pg column (GE Healthcare) equilibrated with buffer A. Fractions of 2 ml were eluted with buffer A and stored at −20°C. Six fractions were selected as representative fractions (F12, 14, 16, 18, 21 and 24) for subsequent analyses.

### 2.2. Negative stain electron microscopy and spirosome length quantification

For visualization of unfractionated 6-His-AdhE, the protein was diluted in buffer B (50 mM tris–HCl pH 8.0, 500 mM NaCl) to a final concentration of 10 µg ml^−1^. To observe the effect of ME0054 compound on 6-His-AdhE, ME0054 stock solution was prepared in dimethyl sulfoxide (DMSO), aliquoted and stored at −20°C in the dark. Fraction 12 (F12) from SEC (2.8 µM) was incubated with ME0054 for 1 h at 37°C at different AdhE : ME0054 molar ratios (1 : 5, 1 : 25 and 1 : 50). As a control without compound (1 : 0), F12 was incubated with the same amount of DMSO (0.14%) used in the sample with the highest compound concentration (i.e. AdhE : ME0054 = 1 : 50) to confirm that any observed effect is not due to the DMSO. After incubation, the samples were diluted 30-fold in buffer B. Drops (5 µl) of each diluted sample were applied to glow-discharged carbon-coated Cu (400 mesh) grids and incubated for 5 min. The grids were washed three times with water and incubated with 2% (w/v) uranyl acetate for 5 min for negative staining. Excess uranyl acetate was removed by filter paper and the grids were dried. Prepared grids were analysed using a JEM-1400Flash transmission electron microscope (TEM) (JEOL) running at 80 keV, images captured using a CDD camera and TEM Center software v. 1.7.26.3016 (JEOL).

For the quantification of spirosome dimensions, the lengths of at least 100 spirosomes from each condition were measured using ImageJ software [18] and analysed using ordinary one-way ANOVA tests.

### 2.3. Small-angle X-ray scattering

SAXS was carried out on beamline B21 of the Diamond Light Source synchrotron facility (Didcot, UK). Data were recorded at 13 keV, at a sample–detector distance of 3.6 m using an Eiger 4M detector (Dectris, Switzerland). For batch mode measurements, 40 µl of AdhE representative fractions (final concentration 1.0 mg ml^−1^) were loaded into a 96-well plate, before being sequentially injected into a quartz capillary by the BioSAXS automatic sample changer (ARINAX). Data were acquired at 6°C and processed with ScÅtter (http://www.bioisis.net) as previously described [12]. SAXS profiles for AdhE spirosome models were computed with the FoXS server [19,20], which was also used to fit models against experimental data (electronic supplementary material, figure S1 and table S1).

### 2.4. Sedimentation velocity analytical ultracentrifugation

Sedimentation velocity AUC (SV-AUC) was performed using a Beckman Coulter XL-I analytical ultracentrifuge equipped with an An-50 Ti eight-hole rotor as previously described [12]. Samples of 300−360 µl (AdhE final concentration 0.28 mg ml^−1^ in buffer A) were loaded into 12 mm pathlength charcoal-filled epon double-sector centrepieces, sandwiched between two sapphire windows and equilibrated at 4°C in vacuum for 6 h before running at 30 000 rpm. To examine the effect of ME0054, AdhE fractions were incubated with ME0054 at different AdhE : ME0054 molar ratios (1 : 0, 1 : 5, 1 : 10, 1 : 25 and 1 : 50, in buffer B) for 1 h at 37°C prior to loading. Absorbance data were recorded in continuous mode between radial positions of 5.65 and 7.25 cm, with a radial resolution of 0.005 cm and a nominal time interval of 7 min. Wavelengths of 280 nm or 330 nm were used to follow AdhE protein or ME0054 compound, respectively. The partial specific volume of AdhE, buffer density and viscosity were calculated using SEDNTERP [21] (electronic supplementary material, table S2). SV-AUC data were analysed using UltraScan-III v. 4 release 6970 [22]. Data were initially edited and then optimized with two-dimensional spectrum analysis (2DSA) [23] to fit the sedimentation boundary, while removing time and radially invariant noise, and fitting the meniscus. 2DSA-Monte Carlo analysis [24] was used to estimate the effect of stochastic noise on the sedimentation and diffusion coefficients prior to further refinement of the fits to the data using parametrically constrained spectrum analysis (PCSA) [25] with Tikhonov regularization. UltraScan calculations were performed on the UltraScan LIMS cluster at the Forschungszentrum Jülich.

### 2.5. Protein fluorophore labelling and total internal reflection fluorescence microscopy

For labelling with Janelia Fluor 549 (JF549) or Janelia Fluor 646 (JF646) fluorophores (emission in the red or far-red part of the visible spectrum, respectively), unfractionated 6-His-AdhE protein in buffer A was dialysed against PBS. Dialysed AdhE diluted in PBSX1 to a final concentration of 3 mg ml^−1^ was incubated with the corresponding fluorophore at a molar ratio of 1 : 1 for 1 h at room temperature (RT) in the dark. After the incubation, the free fluorophore was removed using a Zeba Spin desalting column (0.5 ml) (Thermo Fisher Scientific) following the manufacturer’s instructions.

Equimolar concentrations of protein labelled with JF549 or JF646 were mixed to a final concentration of 0.12 µg ml^−1^, and incubated with ME0054 at different AdhE : ME0054 molar ratios (1 : 0, 1 : 1 and 1 : 10) in PBS1X at 37°C. Every 10 min, 1 µl from the mix was diluted 100-fold in PBS1X.

For imaging, borosilicate glass coverslips (24 mm × 60 mm; VWR, 631-1339) were exposed to argon plasma (Femto, Diener, Germany) for 30 min to remove organic contaminants. 9 × 9 mm^2^ Frame-Seal incubation chambers (Bio-Rad, USA) were affixed to the glass coverslips, and 50 µl of each diluted sample was added to the inside of the chamber and incubated at RT for 20 min. Imaging was performed on a home-built TIRFM microscope. TIRFM restricts detectable fluorescence signal to within 200 nm of the sample slide. For imaging of JF549- and JF646-labelled protein, the outputs from two lasers operating at 561 nm (Cobolt DPL561-100 DPSS Laser System, Cobalt, Sweden) and 638 nm (Cobolt MLD Series 638-140 Diode Laser System, Cobolt, Sweden) were aligned and directed parallel to the optical axis at the edge of a 100×/1.49 numerical aperture TIRFM objective (CFI Apo TIRFM, Nikon, Japan), mounted on an inverted Nikon Ti2 microscope (Nikon, Japan). Fluorescence was collected by the same objective and was separated from the returning TIR beam by a dichroic beamsplitter (Di01-R405/488/561/635, Semrock, USA), and passed through appropriate filters mounted within a motorized filter wheel (561 nm: LP02-568-RS, FF01-587/35, Semrock, USA; 638 nm: FF01-692/40-25, Semrock, USA). Images were recorded on an electron-multiplying charge-coupled device camera (Evolve 512 Delta, Teledyne Photometrics) operating in frame transfer mode (electro-multiplying gain of 11.5 e^−^ per analogue–digital unit (ADU) and 250 ADU per photon). For each data set, 25 images were measured in different regions of the cover slide. Images were recorded at 20 frames s^−1^ for 50 frames, first from the far-red channel (JF646 emission) with 638 nm illumination (37.8 W cm^−2^), followed by 50 frames in the red channel (JF549 emission) with 561 nm illumination (40.6 W cm^−2^). The microscope was automated using the open-source microscopy platform µManager.

Data analysis was performed using custom-written procedures in Python. For each channel, after averaging the stacks over the full set of frames from both channels, the background was first removed by the threshold_local function of the scikit-image Python package (v. 0.20.0). Binary images were then formed by applying a threshold of 2844 for the red channel and 3825 for the far-red channel, and spots corresponding to spirosomes were segmented by applying the measure function of the same Python package. The intensities of the spots in each image corresponded to the maximum brightness in that particular spirosome. The number of coincident events was determined as those that had overlapping intensities in both the red and far-red channels. The number of chance coincident events, which accounts for labelled species that may not actually be associated, but close to each other by chance, was estimated by performing the previously described analyses on red and far-red image stacks, but with the far-red image being first rotated by 90°. The association quotient (*Q*), which is a measure of coincidence, was calculated according to equation (2.1):


(2.1) $$Q=\frac{C-E}{A+B-E},$$

where *Q* is the association quotient, *C* is the number of coincident events in both the red and far-red channels, *E* is the estimated rate at which coincident events occur by chance, *A* is the event rate in the red channel and *B* is the event rate in the far-red channel.

The total intensity of the coincident events was determined by summing the intensities from the red and far-red channels for each spot. These were then binned into histograms of intensity.

The codes used to analyse and plot the data are available at https://doi.org/10.5281/zenodo.11261077 and https://doi.org/10.5281/zenodo.12323461, respectively.

Experiments with labelled AdhE in the absence of ME0054 were performed in triplicate and histograms represent the average of the three replicates including the standard deviation as error bars. Based on the high reproducibility of data acquired in the absence of ME0054, experiments with labelled AdhE in the presence of ME0054 were performed only once.

### 2.6. Enzymatic assays

To determine the enzymatic activity of AdhE fractions (F12 or F24) in the forward and reverse reactions, the consumption or production of NADH, respectively, was measured at a wavelength of 340 nm using a FLUOstar Omega microplate reader (BMG Labtech). All assays were performed at 37°C and the total volume was 100 μl. All reagents were prepared in 50 mM tris–HCl pH 8.0. For measuring the conversion of acetyl-CoA to ethanol (forward reaction), the assays were performed with reaction mixtures containing 50 mM tris–HCl pH 8.0, 250 mM NaCl, 20 μM FeSO_4_, 200 μM acetyl-CoA and 250 μM NADH with 60 μg of AdhE with or without ME0054 at a molar ratio of 1 : 10 (AdhE : ME0054). The conversion of ethanol to acetyl-CoA (reverse reaction) was measured for reaction mixtures containing 50 mM tris–HCl pH 8.0, 250 mM NaCl, 20 μM FeSO_4_, 200 μM CoASH, 200 mM ethanol and 500 μM NAD^+^ with 6 μg of AdhE with or without ME0054 at a molar ratio of 1 : 10 (AdhE : ME0054).

### 2.7. Time-resolved small-angle X-ray scattering

Initial time-resolved (TR)-SAXS characterizing the microfluidic device utilized in this study was conducted on beamline I22 at Diamond Light Source (Oxfordshire, UK) [26]. Addressing the challenges encountered during this initial beamtime, our subsequent experiments (reported here) were conducted on beamline ID02 at the European Synchrotron Radiation Facility (ESRF, Grenoble, France). For the acquisition of data at 37°C, 50 μl of AdhE F12 (final concentration 3.0 mg ml^−1^) with or without ME0054 at a molar ratio of 1 : 10 (AdhE : ME0054) were placed in a quartz capillary of outer diameter 1.1 mm. Nine frames of data from 0.1 s exposures were collected every 3 min over a period of 60 min. The first measurement was taken 4 min after the sample was placed in the capillary.

To carry out TR-SAXS experiments at 25°C, we used a novel microfluidic stopped-flow chip to acquire data every 1 min from 0.06 s, when mixing of the AdhE with ME0054 was complete. Flow chip, engineered open-source fluidic drivers and controllers were designed for beamline use, considering remote operational requirements and X-ray transparency, thereby facilitating robust experimentation and analysis. The vortex T-mixer geometry was optimized using computational fluid dynamics (CFD) simulations, conducted using COMSOL Multiphysics 6.1, with the final device having the highest mixing performance while minimizing dead-time under conditions of lower flow rates when compared with larger-scale counterparts [27]. The final design uses a Duet 3 control board (Duet3D, UK) with stepper motors driving two stages to give precise flow rate control.

For the spirosome perturbation experiments, one syringe contained 1 ml of AdhE F12, while the second was loaded with 0.5 ml of ME0054 (or DMSO as a negative control). The first shot (to fill the tubing between the syringes and the chip) delivered 140 μl from each syringe. Consecutive shots, at a total flow rate of 32.4 ml min^−1^, delivered 42 μl from the syringe containing AdhE and 4.2 μl from the syringe containing ME0054 (at a molar concentration of 100 times that of the protein) to generate a molar ratio of 1 : 10 (AdhE : ME0054) with AdhE at a final concentration of 3.0 mg ml^−1^. Sixty frames of data from 0.05 s exposures were collected every 1 min over a period of 59 min. The acquisition was performed in triplicate.

## 3. Results

### 3.1. Three conserved features in small-angle X-ray scattering profiles are sensitive to AdhE spirosome oligomeric state

**Figure 1.**
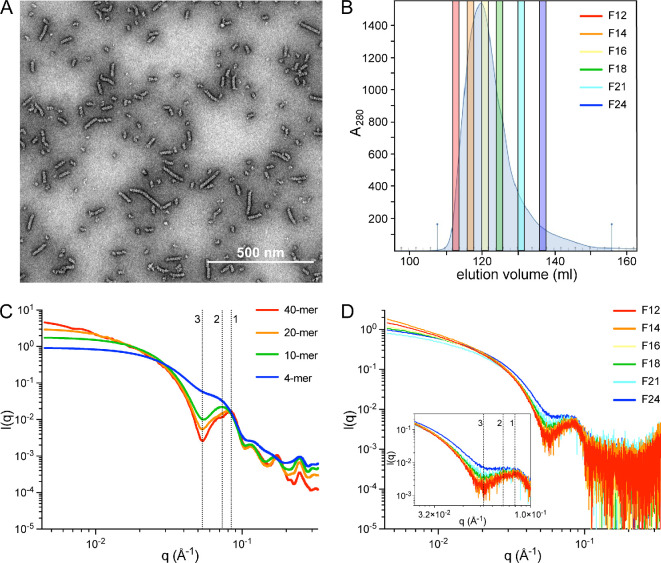
AdhE spirosomes are fractionated by SEC. (A) Unfractionated AdhE spirosomes visualized by negative stain EM. (B) Purified AdhE was fractionated by SEC and six representative fractions (F12–F24) were selected from the elution peak. (C) Small-angle X-ray scattering (SAXS) profiles computed with FoXS server [19,20] for four spirosome models of different lengths. (D) Experimental SAXS data acquired in batch mode for F12–F24 confirming a changing spirosome oligomeric state with elution fraction based on the evolution of the three conserved features, indicated in (C,D) at *q* = 0.086 Å^−1^ (feature 1), *q* = 0.072 Å^−1^ (feature 2) and *q* = 0.054 Å^−1^ or *q* = 0.057 Å^−1^ (feature 3, in computed and experimental profiles, respectively).

AdhE with a 6-His tag fusion at the N-terminal was purified by immobilized metal affinity chromatography. As previously described [12,14], negative stain electron microscopy (EM; figure 1A) revealed that purified AdhE formed spirosomes heterogeneous in length. AdhE spirosomes were fractionated (by SEC) based on their hydrodynamic volume. Six 2 ml fractions (termed F12, F14, F16, F18, F21 and F24) were collected across the elution peak (figure 1B). SAXS data for the AdhE fractions were obtained in batch mode. Previous work [12] correlated a conserved feature in AdhE spirosome SAXS profiles at reciprocal space position *q* = 0.086 Å^−1^ with the spirosome cryo-EM structure helical pitch. We hypothesized that loss of helical repeats per particle when spirosome length decreases would be reflected in a change in this conserved feature. To test this, SAXS profiles were computed with FoXS [19,20] for linear spirosome models (4-, 10-, 20- and 40-mer; figure 1C), constructed from the atomic resolution structure of AdhE (PDB ID 6AHC [12]). As expected, a dramatic shift in the position of the conserved feature was observed with reduction in spirosome model length from 40-mer (*q* = 0.086 Å^−1^) to 4-mer (*q* = 0.072 Å^−1^) (features 1 and 2; figure 1C), supporting our hypothesis. Loss of helical repeats was also reflected in a gradual reduction in the low-angle (*q* < 0.025 Å^−1^) slope, consistent with a lower radius of gyration corresponding to shorter spirosomes. Interestingly, a very pronounced trough at *q* = 0.054 Å^−1^ diminished progressively with the reduction in length, becoming a new indicator for spirosome length analysis (feature 3; figure 1C).

Comparison of experimental data (figure 1D) with profiles computed for the four spirosome models (figure 1C) confirms that the earliest eluting faction (F12) is dominated by large oligomers while fractions collected thereafter comprise increasingly shorter spirosomes. To estimate the average spirosome length in each fraction, data were fitted with FoXS [19,20] for a wider series of models. The oligomeric states providing the best fits for each fraction (electronic supplementary material, table S1) reflect a decrease in observable dominant spirosome length from F12 to F24 of 40-mer to 14-mer.

### 3.2. Sedimentation velocity analytical ultracentrifugation reveals that size exclusion chromatography fractionation of AdhE is only partial

**Figure 2.**
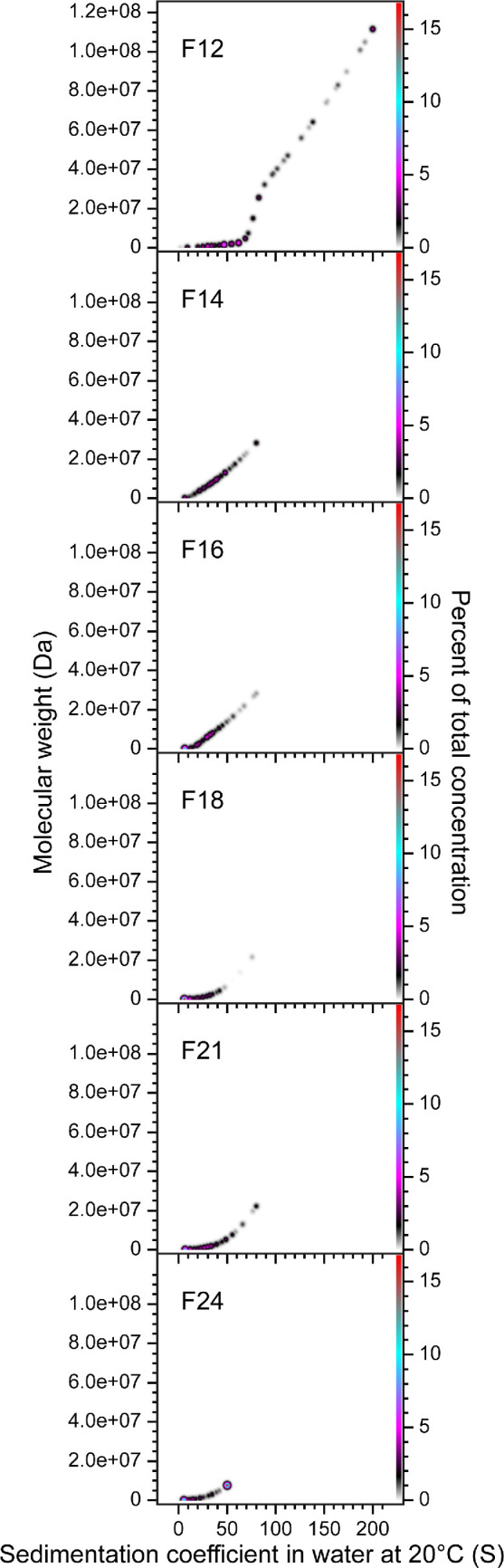
SV-AUC reveals that SEC fractionation of AdhE is only partial. The oligomeric state of six representative AdhE SEC fractions (F12–F24) was determined by PCSA of SV-AUC data, which revealed that fractionation is only partial. The SV-AUC data were fitted with an increasing sigmoid (IS) function; Tikhonov regularization was used to provide limited data smoothing. F12, the earliest eluting fraction, includes spirosomes with oligomeric states from dimer to 1000-mer. The highest oligomeric state in F24, the latest eluting fraction, is approximately 75-mer.

SEC fractions F12 to F24 were analysed by SV-AUC, which revealed that none of the fractions was homogeneous, with all PCSA comprising multiple discrete species (figure 2) indicating that the spirosomes were only partially fractionated by SEC. The PCSA spectra for later eluting fractions are increasingly dominated by smaller species, consistent with a reduction in oligomeric state. The molecular weight of monomeric AdhE is 96.1 kDa. Thus, F12, the earliest eluting fraction, includes oligomeric states from dimer to 1000-mer. The highest oligomeric state in F24, the latest eluting fraction, is approximately 75-mer. In heterogeneous samples, SAXS is dominated by scattering from bigger species. Thus, although later eluting fractions, such as F24, are shown by SV-AUC to be dominated by smaller species (figure 2), the presence of larger oligomers will result in elevated values of χ^2^ for the fit to the experimental data by the models, as observed in electronic supplementary material, figure S1 and table S1.

### 3.3. AdhE spirosome formation is dynamic in the absence of stimuli

SAXS and SV-AUC revealed it was not possible to fully fractionate AdhE spirosomes by SEC (figures 1 and 2; electronic supplementary material, figure S1 and table S1). This could be explained if spirosome formation is dynamic in the absence of stimuli, in which case, after fractionation, spirosomes could assemble and/or disassemble. To explore this further, we used single-molecule microscopy to visualize individual spirosomes, exploring their dynamics over time. Similar to previous studies [28,29], equimolar concentrations of AdhE were labelled with two different fluorophores and TIRFM was used to characterize protein exchange between individual AdhE complexes. It was expected that upon mixing of the two differently labelled samples, dynamic exchange of subunits between spirosomes would result in spirosomes labelled with two differently coloured fluorophores.

**Figure 3.**
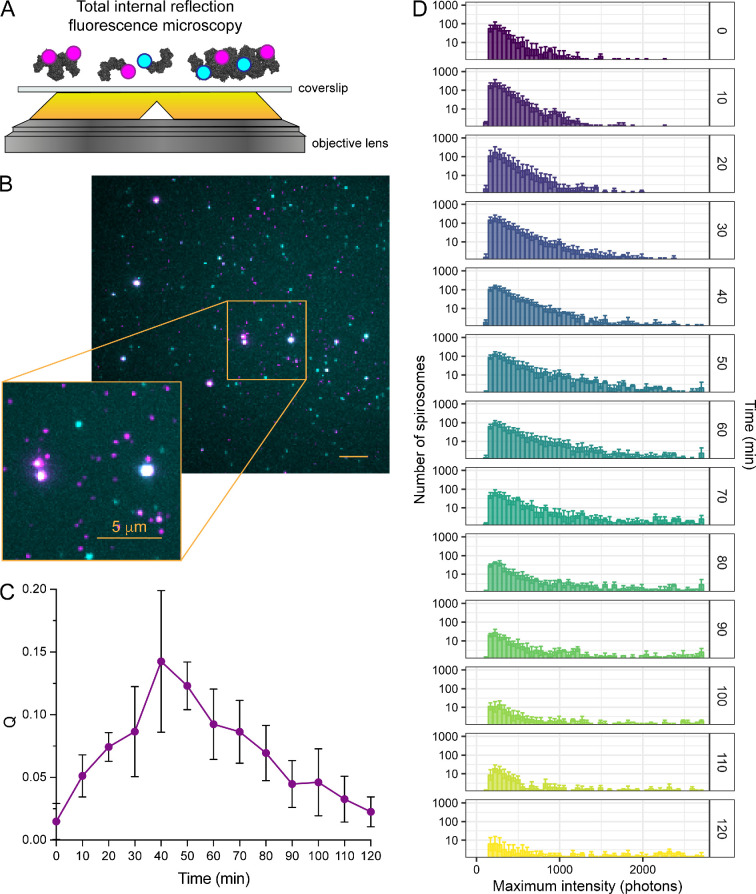
AdhE spirosome formation is dynamic in the absence of stimuli. (A) Schematic diagram of TIRFM setup. Equimolar concentrations of Janelia Fluor 646-labelled (cyan) and Janelia Fluor 549-labelled (magenta) unfractionated spirosomes incubated at 37°C were imaged by TIRFM over 120 min. (B) Representative TIRFM merged image after 70 min incubation at 37°C showing coincidence (in white) of the two fluorophores on the same spirosome as a consequence of AdhE spirosome formation. (C) Plot of association quotient (*Q*) over the time course of the experiment as a measurement of the fraction of new molecules containing both fluorophores. (D) Histograms of dual-labelled spirosome fluorescence intensity over the experiment time course, where shorter spirosomes correlate with lower intensity values and larger spirosomes correlate with higher intensity values. As time progressed, the formation of larger spirosomes was observed (as indicated by an increase in the number of high-intensity spirosomes). In (C,D), error bars represent the standard deviation.

Equal concentrations of unfractionated AdhE labelled with either Janelia Fluor 549 (JF549) or Janelia Fluor 646 (JF646) were mixed and incubated at 37°C. From this mixture, samples were imaged by TIRFM over 120 min (figure 3A,B; electronic supplementary material, figure S2). At the start of the incubation, the association quotient (*Q*, a measure of the fraction of molecules containing both fluorophores) was low (figure 3C). After further incubation, the number of spirosomes containing both JF549 (magenta in figure 3B; electronic supplementary material, figure S2) and JF646 (cyan in figure 3B; electronic supplementary material, figure S2) increased, resulting in an increase in *Q* up to 40 min. Subsequently, *Q* decreased, which could reflect (a) spirosome disassembly and/or (b) spirosome length increase, e.g. through continued association of double-labelled spirosomes. When the intensities of individual spirosomes containing both fluorophores were binned into histograms, there was a clear increase in the proportion of higher intensity, and thus longer, spirosomes (figure 3D; electronic supplementary material, video S1c), supporting (b) as the primary source of increased *Q*. This time-dependent effect was also observed when intensities of individually labelled spirosomes were measured (electronic supplementary material, figures S3 and S4, video S1a,b). Overall, these data suggest that AdhE spirosomes assemble dynamically in the absence of stimuli, but we are unable to determine from these data whether they also disassemble.

### 3.4. AdhE spirosome length drives AdhE enzymatic reaction direction

**Figure 4.**
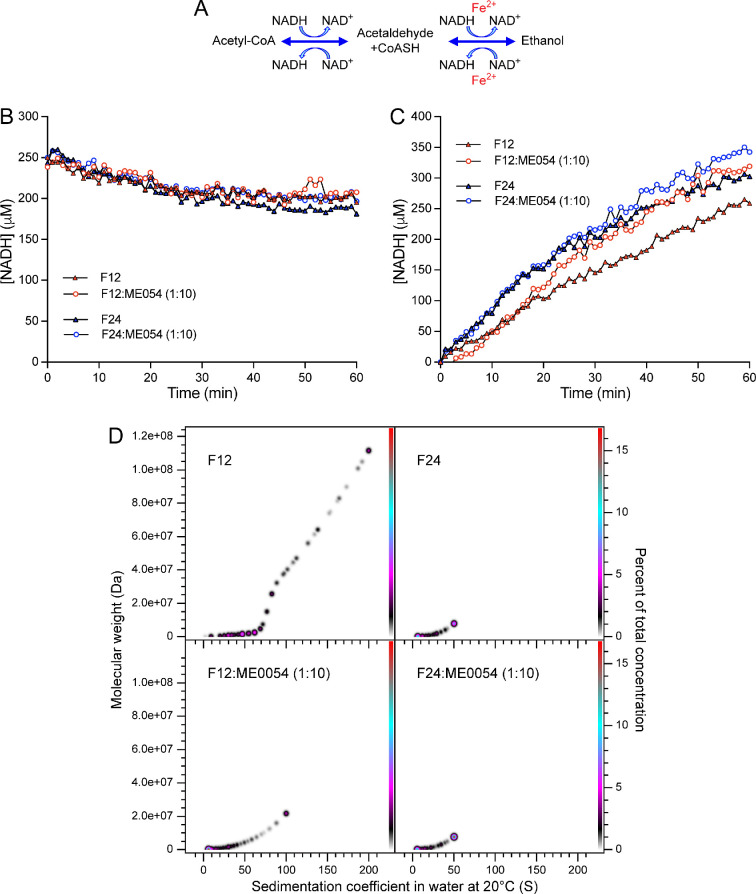
AdhE spirosome length drives the direction of the enzymatic reaction. (A) Schematic representation of the enzymatic reactions catalysed by AdhE. Enzymatic assay of the forward (B) or reverse (C) reactions catalysed by AdhE. For (B,C), AdhE fractions contained a main population of long (F12, red symbols) or short (F24, blue symbols) spirosomes in the presence (open circles) or absence (filled triangles) of ME0054 (1 : 10 molar ratio (AdhE : ME0054)). (D) PCSA analysis of SV-AUC data for F12 or F24 incubated for 1 h ± ME0054 (1 : 10 molar ratio (AdhE : ME0054)) at 37°C. SV-AUC data were fitted with an increasing sigmoid (IS) function; Tikhonov regularization was used to provide limited data smoothing.

AdhE converts acetyl-CoA to ethanol (forward reaction) and ethanol to acetyl-CoA (reverse reaction; figure 4A) [11–14]. To explore whether spirosome length is related to enzymatic efficiency, the activity of F12 and F24 (dominated by long and short spirosomes, respectively) was tested in the forward and reverse reactions. To measure the forward reaction, F12 or F24 was incubated with acetyl-CoA in the presence of NADH, and activity was measured by monitoring NADH consumption at 340 nm. No difference in activity was observed between F12 and F24, suggesting that spirosome length has no impact on AdhE activity in the forward reaction (figure 4B). To measure the reverse reaction, F12 or F24 was incubated with ethanol, NAD^+^ and CoASH activity was measured by monitoring NADH production at 340 nm. Surprisingly, in the reverse reaction, the fraction dominated by short spirosomes (F24) was much more efficient than that dominated by long spirosomes (F12; Figure 4C). These results suggest that AdhE needs to form spirosomes to balance the direction of enzymatic reaction.

### 3.5. ME0054 binds to AdhE spirosomes and perturbs them in a concentration-dependent manner

Previous studies proposed AdhE as an SA compound target in EHEC [11]. To examine whether SA compounds affect AdhE enzymatic activity, F12 and F24 were incubated with the SA compound ME0054 at a 1 : 10 molar ratio (AdhE : ME0054) during the forward and reverse reactions (figure 4B,C). No impact on AdhE activity was observed in the presence of ME0054 during the forward reaction (figure 4B). However, in the reverse reaction, the efficiency of long spirosomes (F12) dramatically increased after 17 min in the presence of ME0054, reaching a level comparable to that attained by short spirosomes (F24; figure 4C). In contrast, the activity of F24 (dominated by shorter spirosomes) only slightly increased in the presence of ME0054 (figure 4C). These data suggest that ME0054 perturbs AdhE spirosomes, transforming the oligomeric profile of F12 to something resembling F24.

To explore this idea, F12 and F24 were incubated with ME0054 for 1 h at 37°C at a molar ratio of 1 : 10 (AdhE : ME0054) and analysed by SV-AUC (figure 4D). The PCSA spectrum for F12 shifted towards lower sedimentation coefficients and molecular weights in the presence of ME0054, with the loss of spirosomes with oligomeric state greater than 250-mer and a dramatic shift in the population density towards species of 50-mer or less, confirming that ME0054 perturbs AdhE spirosomes. When a sample dominated by short spirosomes (F24) was incubated with ME0054, the change in PCSA spectrum was imperceptible (figure 4D), since the sample contained no large (i.e. *n* > 75mer) oligomers to perturb.

**Figure 5.**
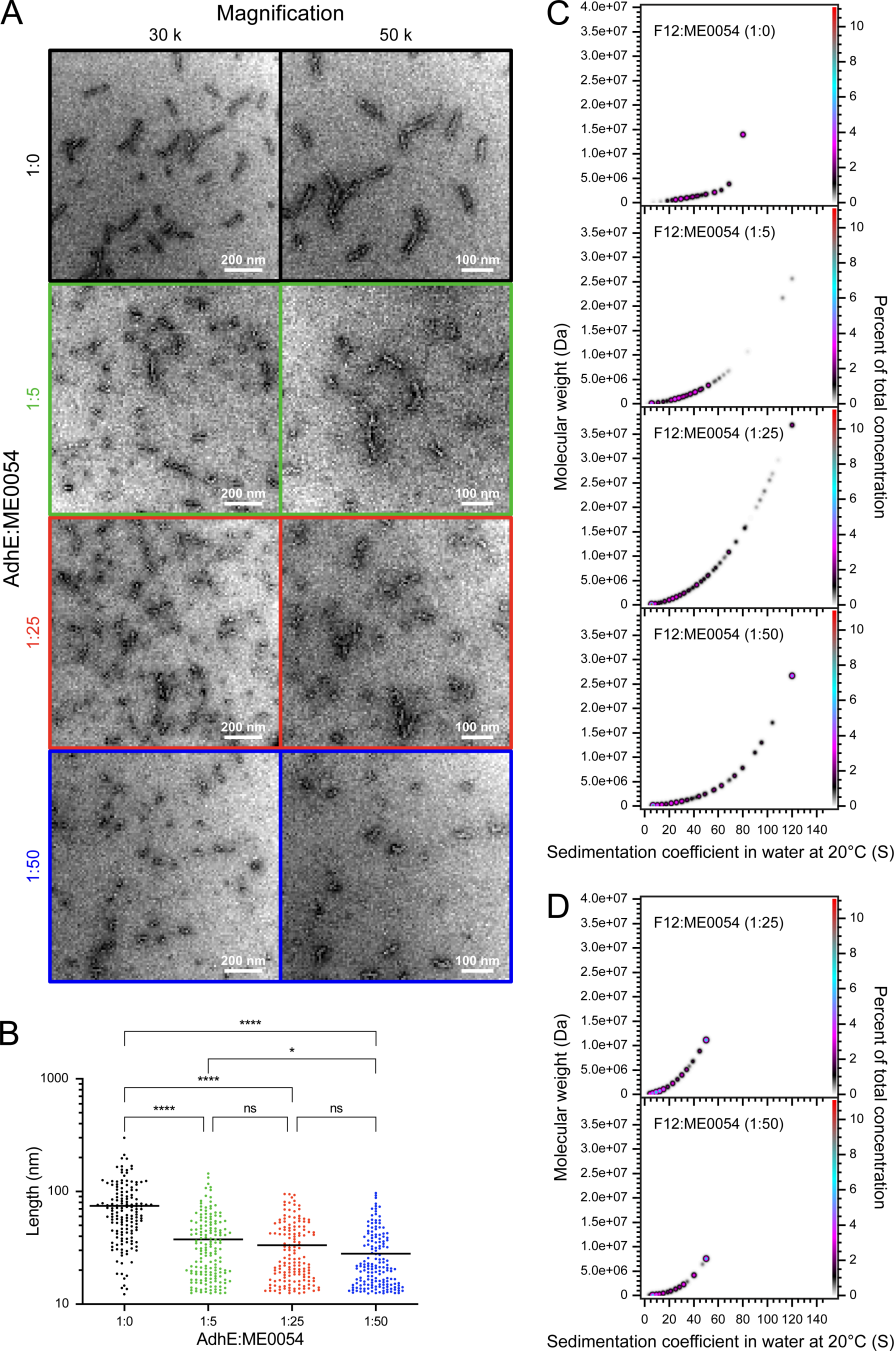
ME0054 binds to AdhE spirosomes and perturbs them in a concentration-dependent manner. AdhE fraction 12 (F12), dominated by long spirosomes, was incubated for 1 h at 37°C with ME0054 at different (1 : 0, 1 : 5, 1 : 25 and 1 : 50 (AdhE : ME0054)) molar ratios. In (A,B), black signifies an AdhE : ME0054 molar ratio of 1 : 0, green 1 : 5, red 1 : 25 and blue 1 : 50. (A) Perturbation of AdhE spirosomes by ME0054 was visualized by negative stain electron microscopy (EM) at magnifications of 30 000× and 50 000×. (B) The lengths of at least 100 spirosomes from each condition were measured using ImageJ software [18] (*n* ≥ 100, ^ns^ not significant, *p < 0.05, ****p < 0.0001, one-way ANOVA, mean (horizontal black line)). (C) PCSA analysis of SV-AUC data acquired with absorbance optics at 280 nm for observation of sedimenting protein for AdhE F12 in which the data were fitted with an IS function. (D) PCSA analysis of SV-AUC data acquired with absorbance optics at 330 nm for observation of the chromophore on ME0054 for AdhE F12 in which the data were fitted with an IS function. In (C,D), Tikhonov regularization was used to provide limited data smoothing.

To further investigate AdhE spirosome perturbation by ME0054, F12 was incubated with ME0054 at different (AdhE : ME0054) molar ratios for 1 h at 37°C for visualization of the effect by negative stain EM (figure 5A). The lengths of at least 100 molecules per condition were quantified (figure 5B), concluding that the impact of ME0054 on AdhE spirosomes is concentration dependent. To confirm that small molecules were not missed during quantification due to limits of microscope magnification, the same conditions were tested by SV-AUC at 280 nm (figure 5C) and 330 nm (figure 5D) to observe AdhE and ME0054, respectively. SV-AUC data recorded at 280 nm support the conclusion that ME0054 perturbs AdhE spirosomes in a concentration-dependent manner, since with increasing AdhE : ME0054 molar ratios, there is an increase in the proportion of smaller species in the PCSA spectra (figure 5C). The SV-AUC data recorded at 330 nm show that the compound binds to AdhE, because, at both AdhE : ME0054 molar ratios observed, ME0054 co-sedimented with spirosomes (figure 5D). These data also demonstrate that the high molecular weight, high sedimentation coefficient species that begin to appear with increasing concentrations of ME0054 (figure 5C) do not have compound bound to them.

The concentration-dependent impact of the compound on unfractionated AdhE at different time points was observed using TIRFM. Equal concentrations of unfractionated AdhE labelled with JF549 or JF646 were mixed and incubated at 37°C with ME0054 at different AdhE : ME0054 molar ratios (1 : 0, 1 : 1 and 1 : 10). Fluorophores were tracked individually (electronic supplementary material, figures S3 and S4 and videos S2 and S3) and coinciding on the same spirosome (electronic supplementary material, figure S5 and video S4). The results confirm that ME0054 continues to perturb spirosome assembly 1 h after addition to AdhE, based on the decrease in appearance of ‘high intensity’ (longer) spirosomes with time in the presence of the compound (electronic supplementary material, figures S3–S5 and videos S2–S4).

### 3.6. ME0054 perturbs AdhE spirosomes within four minutes

**Figure 6.**
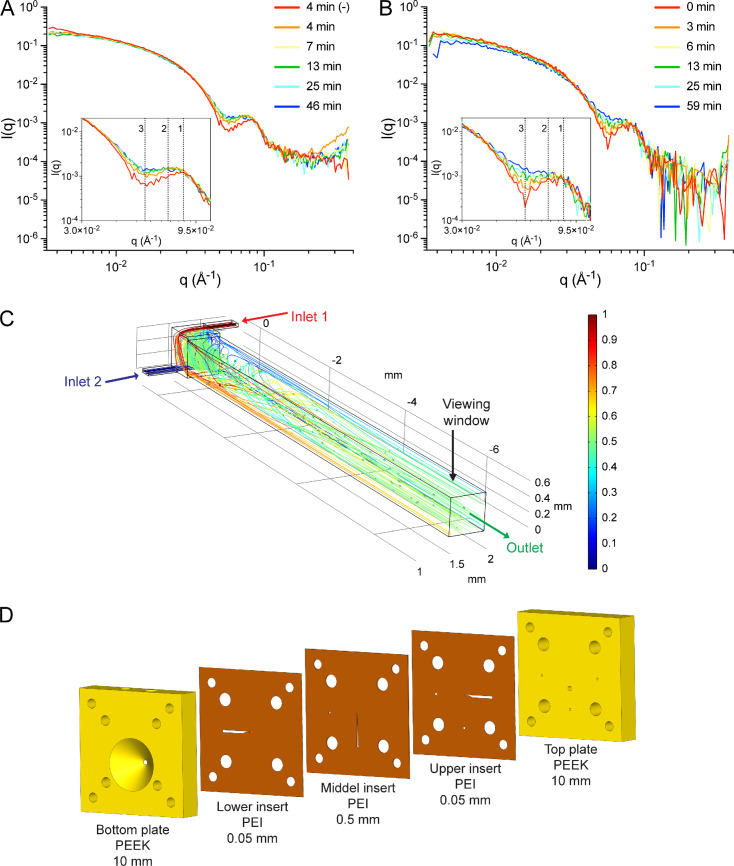
ME0054 perturbs AdhE spirosomes within 4 min. AdhE fraction 12, dominated by long spirosomes, was mixed with ME0054 at a molar ratio of 1 : 10 (AdhE : ME0054) for the acquisition of SAXS data (A,B). The evolution of features at *q* = 0.086 Å^−1^ (feature 1) to *q* = 0.072 Å^−1^ (feature 2) together with a diminution of that at *q* = 0.057 Å^−1^ (feature 3) is evident, consistent with a loss of the spirosome helical repeat. (A) Data were acquired for pre-mixed sample in a quartz capillary at 37°C every 3 min for 1 h. (B) Data were acquired for sample mixed with a novel X-ray transparent microfluidic stopped-flow chip (C,D) at 25°C every 1 min for 1 h. For (A,B) for clarity, only data for selected time points are shown. (C) Depiction of a vortex T-mixer and the CFD simulation results (COMSOL Multiphysics 6.1) showcasing coloured streamlines corresponding to concentration and the viewing window. This simulation informed the optimization process for enhanced mixing performance and minimized dead time. (D) Drawing of the layered constructed microfluidic chip (SOLIDWORKS 2022) incorporating the optimized vortex T-mixer design.

Computation of SAXS profiles for linear AdhE spirosome models (figure 1C) confirmed that it is possible to distinguish between and determine dominant spirosome lengths in an AdhE fraction based on SAXS data (figure 1D). We used TR-SAXS to explore the kinetics of spirosome perturbation by ME0054. In contrast to TIRFM, TR-SAXS allowed us to acquire data within shorter time periods. AdhE F12 was mixed with ME0054 at a molar ratio of 1 : 10 (AdhE : ME0054), and SAXS data were recorded every 3 min from 4 min for 60 min at 37°C (figure 6A). Due to limitations imposed by the beamline environment, it was not possible to acquire data at this temperature before 4 min. Comparison of the data obtained after 4 min at 37°C in the presence or absence of ME0054 clearly shows changes in features at *q* = 0.086 Å^−1^ and *q* = 0.057 Å^−1^ (figure 6A) consistent with a reduction in spirosome length in the presence of ME0054. In contrast to static measurements (figure 1D), no apparent changes were detected at low angles (*q* < 0.025 Å^−1^), owing to the shorter camera length used (2 m, compared with 3.6 m). After 4 min, changes to profiles recorded in the presence of ME0054 became more subtle, suggesting that most of the effect of the compound was produced by then. To follow spirosome perturbation dynamics with enhanced time resolution, we decreased the temperature to 25°C, expecting that the process might be slowed and employed a novel X-ray transparent microfluidic stopped-flow chip. This chip differs from traditional stopped-flow devices by integrating a vortex T-mixer design (figure 6C), which has a higher mixing efficacy than standard T-mixers [30]. Figure 6C shows a sample simulation result at a total flow rate of 3 ml min^−1^, using coloured streamlines corresponding to the concentrations of two inlet streams, 1 (red) and 0 (blue), and the subsequent mixing towards a concentration of 0.5 (green). Based on the simulation results, a layered fabrication approach, inspired by Levenstein *et al.* [31], was adopted to construct the chip shown in figure 6D using a mixture of laser-cutting and computer numerical control machining. Data were acquired every 1 min from 0.06 s, when mixing of AdhE with ME0054 was completed. The new microfluidic stopped-flow chip allowed us to minimize sample volumes, maximize the number of data recorded and significantly reduce dead time at the start of data acquisition. We managed to observe the evolution of key SAXS profile features in greater detail and over a more extended period (59 min) as the result of the perturbation of AdhE spirosomes by ME0054 at 25°C (figure 6B). These results confirm that, at biologically relevant temperatures, ME0054 perturbs AdhE spirosomes within 4 min and that this perturbation continues for at least 55 min thereafter.

## 4. Discussion

The bacterial enzyme AdhE presents a captivating enigma on multiple fronts. It forms spirosomes, crucial for its activity, but the requirement for such a diverse range of spirosome lengths remained a mystery. This fundamental gap in understanding the structure–function relationship of AdhE has impeded our understanding of how this protein impacts bacterial metabolism. AdhE is a bidirectional aldehyde–alcohol dehydrogenase. Its N-terminal aldehyde dehydrogenase (ALDH) domain converts acetyl-CoA to acetaldehyde, a toxic intermediate. The acetaldehyde is converted to ethanol by the AdhE C-terminal alcohol dehydrogenase (ADH) domain [12]. Initially, we replicated previous results [14] showing by negative stain EM that purified AdhE with a 6-His tag fusion at the N-terminal can oligomerize, forming helicoidal filaments heterogeneous in length called spirosomes. Why might it be advantageous to the parent organism to vary the length of AdhE spirosomes? We envisioned two hypotheses to explain this: (a) longer spirosomes are enzymatically more efficient or (b) spirosome length drives enzymatic reaction direction. It is plausible that a long spirosome contains a higher number of ALDH and ADH domains compared with a short one and consequently could be enzymatically more efficient, simply by virtue of the availability of an increased number of domains nearby able to accept substrate. The high-resolution cryo-EM structure of one-and-a-half spirosome helical turns showed that the ALDH domains are located towards the outer surface of the helical structure, whereas the ADH domains point towards the inner surface [12]. Thus, the two enzymatic activities are topologically separated in the spirosome [12]. Additionally, variation in spirosome length generates filaments with different accessibilities to the ALDH and ADH domains, which would be more or less efficient in one direction of the reaction, and thus differences in spirosome lengths would be necessary to control the direction of the enzymatic reaction carried out by AdhE. To explore these options, in this work, AdhE spirosomes were fractionated by size using SEC (figure 1B) to compare the enzymatic efficiency of long versus short spirosomes in the two directions of the reaction (figure 4B,C).

During SEC fractionation, AdhE eluted in a single asymmetric peak (figure 1B), so fractions were collected during elution and analysed by SAXS and SV-AUC. An important difference between SAXS and SV-AUC is that the size of species resolvable by the former is dependent on the X-ray energy and camera length used, while for the latter it is dependent on rotor speed and scan interval. At the SAXS camera length and X-ray energy used in this work (3.6 m, 13 keV), particle sizes that can be reliably observed range from 10 to 1000 Å, covering monomer to 54-mer. Whereas at the AUC rotor speed (30 000 rpm) and scan interval (nominally 7 min) used, species with sedimentation coefficients of 5−200 S are observable, corresponding to monomer to 1000-mer. SAXS data (figure 1D) could be recapitulated (figure 1C) by linear spirosome models of differing lengths, three features diagnostic of the spirosome helical repeat evolving with fraction number. PCSA [25] was chosen as the most suitable tool with which to analyse SV-AUC data because it simultaneously models heterogeneity in size and anisotropy of macromolecular mixtures and is suited to polymerizing systems exhibiting a systematic size and shape growth function. PCSA of SV-AUC data (figure 2) confirmed that AdhE was only partially fractionated by SEC, suggesting that spirosome formation could be a dynamic process: although the spirosomes could be initially fractionated based on hydrodynamic volume, they may re-equilibrate to broader oligomeric distributions after elution. This possibility was examined by TIRFM, where spirosome formation was observed in the absence of stimuli (figure 3; electronic supplementary material, figure S2 and video S1). By labelling unfractionated AdhE samples with two different fluorophores and then mixing them (figure 3A), it was possible to observe the increase over time in the number of statistically significant coincidences between the fluorophores (figure 3C), which occurs only when they are co-located on the same spirosome (figure 3B). This would happen when differently labelled spirosomes join to form a longer spirosome, which is evident in the increase in species with greater maximum intensities (figure 3D) over time. This confirms the propensity of AdhE spirosomes to continue to form an expanded repertoire of oligomeric species, even without an external stimulus.

To understand why AdhE forms spirosomes with different lengths, fractions containing a main population of long (F12) or short (F24) spirosomes were generated and enzymatic assays were performed to compare activities in the forward and reverse reactions (figure 4A*–*C). Spirosome length did not have any impact in the forward reaction (figure 4B). However, short spirosomes were more efficient than long during the reverse reaction (figure 4C), refuting hypothesis (a). When the spirosome is short, the ADH domains on the inner surface of the filament are more accessible to the ethanol substrate than they are in a longer spirosome. In contrast, the disposition of the ALDH domains towards the spirosome outer surface makes them constantly accessible to the acetyl-CoA substrate, independent of spirosome length. The observation that converting acetyl-CoA to ethanol (forward reaction) has the same efficiency independent of spirosome length (figure 4B) could be relevant for industrial biofuel production. Our results demonstrate that controlling spirosome length would be unnecessary for ethanol production by AdhE, simplifying the process and making it possible for unfractionated protein to be used as the biological catalyst.

**Figure 7.**
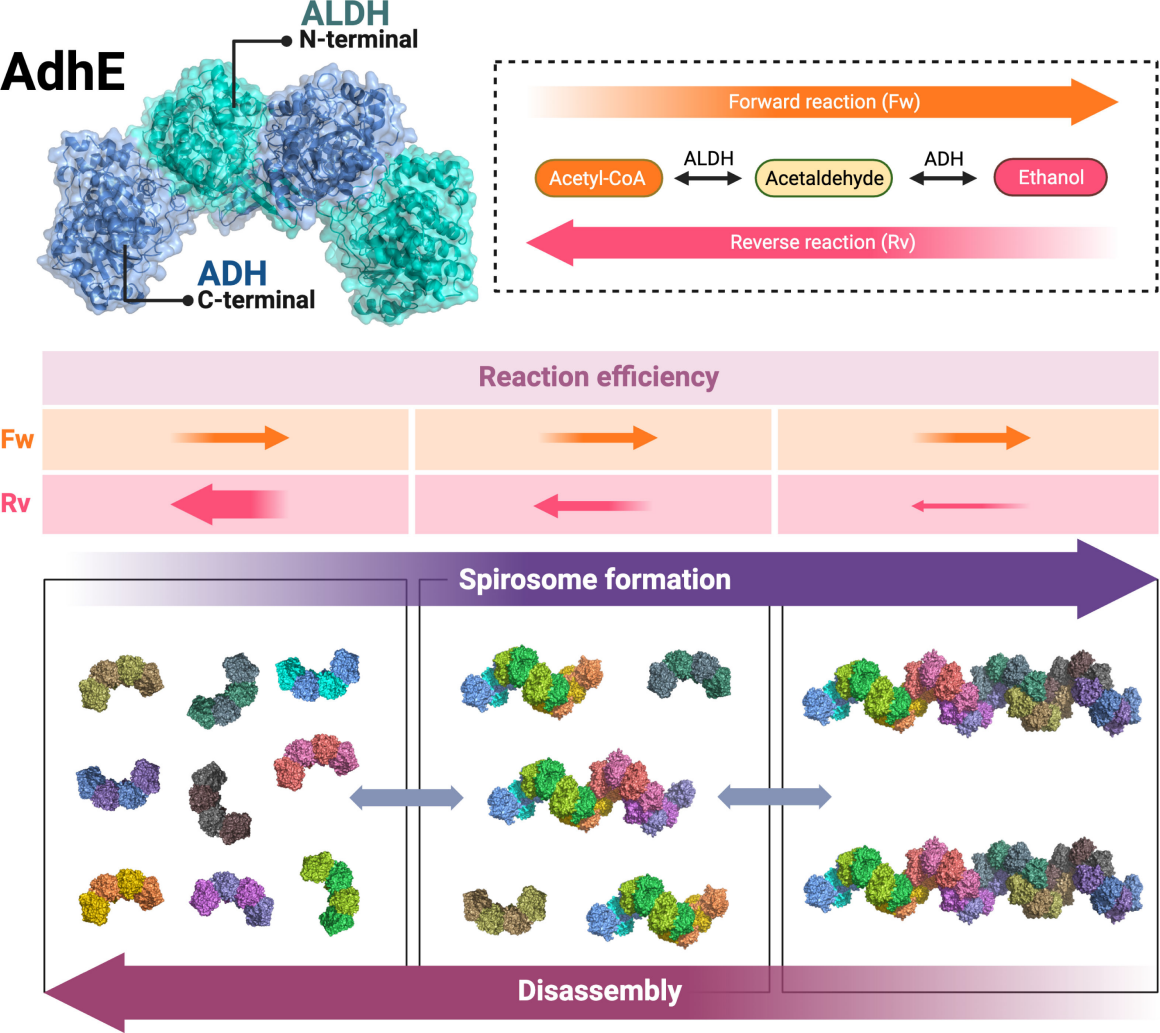
Model explains that AdhE forms spirosomes to control the direction of its enzymatic reactions. *Top*: AdhE dimer model and schematic representation of AdhE enzymatic activity in the forward (Fw) and reverse (Rv) reactions. *Bottom*: three scenarios showing short, long or a mix of spirosome lengths during AdhE spirosome formation or disassembly. *Middle*: the forward (Fw) reaction efficiency is constant in the different scenarios whereas short spirosomes are more efficient during the reverse (Rv) reaction. Created with BioRender.com.

The fact that short spirosomes are more efficient than long spirosomes in the conversion of ethanol to acetyl-CoA allows us to propose the following models to explain the relationship between spirosome length and enzymatic reaction direction (figure 7). In a situation where only short spirosomes are present, the efficiency of the reverse reaction would be very high, reducing the levels of ethanol and accumulating acetyl-CoA in the bacterial cytoplasm. AdhE spirosome formation would be necessary to decrease the efficiency of the reverse reaction (figure 7). Because the efficiency of the forward reaction is constant, in an intermediate situation with a mix of short and long spirosomes, both directions of the reaction are balanced (figure 7). Taken together, these results suggest that AdhE spirosome formation, coupled with dynamic disassembly of the filaments, is necessary to control the direction of the enzymatic reaction carried out by AdhE, supporting hypothesis (b).

The underlying mechanism between AdhE structure–function and expression of the T3SS has yet to be understood. However, this ‘Achilles heel’ provides a weakness that may be exploited to interfere with the pathogen’s capacity to attach to host cells successfully. Therefore, inhibitors that perturb AdhE structure and function are of interest as potential anti-infective or ‘anti-virulence’ agents. To this end, understanding the mechanism by which the SA compounds affect AdhE was a further goal of this work. In this study, we formally confirm the direct effect of an SA compound (ME0054) on AdhE spirosomes. The enzymatic activities of fractions containing a main population of long (F12) or short (F24) spirosomes were tested in the presence of ME0054 (figure 4B,C). Spirosome activity increased in the presence of ME0054 during the reverse reaction (figure 4C), whereas no effect on activity was observed in the forward reaction (figure 4B). Because short spirosomes are more efficient in the reverse reaction this result suggests that ME0054 could be perturbing the spirosomes and therefore enhancing the efficiency of the reverse reaction. To confirm this, AdhE F12, comprising a main population of long spirosomes, was incubated with ME0054 at different AdhE : ME0054 molar ratios. Data from negative stain EM showed that ME0054 perturbed AdhE spirosomes in a concentration-dependent manner (figure 5). However, a limitation of the methodology was that the smallest molecules could not be detected by negative stain EM due to the magnification scale. This explains why the ordinary one-way ANOVA tests showed that the differences in length were statistically significant only when the control without ME0054 was compared with different compound concentrations (figure 5B). Therefore, to quantify all molecules present in the sample, SV-AUC was performed by measuring the absorbance at 280 nm to follow AdhE spirosomes. We observed how the PCSA spectrum for the sample without ME0054 was displaced to lower values of s_20,w_ and molecular weights in the presence of compound (figure 5C), directly correlating with a reduction in spirosome size, confirming the observation from negative stain EM that an increase in compound concentration resulted in greater spirosome perturbation and species. In addition, the effect of ME0054 on unfractionated AdhE spirosomes at different AdhE : ME0054 molar ratios was tracked with time by TIRFM (electronic supplementary material, figures S3–S5 and videos S2–S4) confirming that the compound continues to perturb the spirosome length after 1 h.

To examine whether the compound binds to the spirosome or perturbs and then dissociates from it, SV-AUC data were acquired at a wavelength of 330 nm (figure 5D) at which ME0054, but not AdhE, absorbs. It was not possible to properly analyse the SV-AUC data acquired at 330 nm at an AdhE : ME0054 molar ratio of 1 : 5 since the signal was too weak. The molecular weight of ME0054 is 272 Da and as such, it would be expected to sediment with s_20,w_ ~ 0.2 S; however, the PCSA spectra arising from analysis of the SV-AUC data recorded at 330 nm comprise species ranging in molecular weight from ~100 kDa to 7.5 MDa and in sedimentation coefficient from ~6 S to 50 S, consistent with monomeric to 78-mer AdhE, confirming co-sedimentation of and thus binding of ME0054 with AdhE. The appearance of very low concentrations (1% of total concentration) of high molecular weight, high sedimentation coefficient species as concentrations of ME0054 increase (figure 5C) may result from the release of small protomers from perturbed spirosomes which are then available to join to existing spirosomes, forming the larger observable species.

Finally, the effect of ME0054 on long spirosomes (F12) was tested at 37°C and 25°C by TR-SAXS (figure 6). Conventional SAXS was able to provide data only 4 min after sample mixing. Thus, in order to acquire data to report on the very initial stages of the perturbation process and to do so with minimal (50 µl) sample requirements, a novel X-ray transparent microfluidic stopped-flow chip was designed (figure 6C,D), optimized and employed (figure 6B). As expected, the compound acted more rapidly at 37°C (figure 6A) than at 25°C (figure 6B), but was nonetheless still active at 25°C. This is an exciting and first demonstration of this new microfluidic stopped-flow chip.

AdhE spirosomes perturbed by ME0054 are shorter and thus more efficient in the reverse reaction (figures 4C,D and 5D). Therefore, the addition of ME0054 enhances the consumption of ethanol and increases the accumulation of acetyl-CoA in the bacterial cytoplasm. Within the cell acetyl-CoA can be converted to acetyl-phosphate and acetate, so we would expect both of these metabolites to be increased. Intriguingly, previous studies have shown that deletion of *adhE* results in a 20% increase in acetate secretion compared with the wild-type strain [11]. Hence, our finding that ME0054 perturbs AdhE spirosomes, thereby favouring the accumulation of acetyl-CoA, may provide an explanation for the SA compounds mirroring some of the phenotypes generated by deletion of *adhE*, including a reduction in virulence by inhibition of the T3SS [11,17]. However, even though in this work AdhE has been validated as a protein target of ME0054, we acknowledge the complexity caused by the finding that ME0054 interacts with other proteins [10], as these interactions will invariably contribute to the observed bacterial phenotypes.

Taken together, these results confirm that AdhE is indeed a protein target for ME0054 and that the compound acts by perturbing AdhE spirosomes. This step forward in our understanding of how the SA family of compounds works in EHEC will help in the design of more specific and targeted inhibitors of AdhE that have greater stability and selectivity.

## 5. Opening up

New treatments and approaches to target bacterial infections are one of the most important health challenges of the modern era. There are no easy solutions to this problem, and our ongoing work aims to address long-standing unanswered questions about fundamental aspects of the role played by the bidirectional, bifunctional enzyme AdhE in bacterial metabolism and virulence. AdhE presents an exciting and highly conserved target utilized by a suite of Gram-negative pathogens. Our multidisciplinary approach has, for the first time, revealed that AdhE spirosome formation is necessary to regulate the direction of its enzymatic reactions and that a SA binds to and disrupts spirosomes at biologically relevant temperatures and timescales, thereby enhancing conversion of ethanol to acetyl-CoA. The mechanism of action of SA in this regard remains formally undetermined. Based on the work reported here, we hypothesize that the enhanced accumulation of acetyl-CoA due to spirosome disassembly causes protein acetylation, resulting in perturbation of bacterial metabolism.

The impact of SA compounds on bacterial virulence has been extensively demonstrated in the Gram-negative species *Yersinia*, *Chlamydia*, *Salmonella*, *Shigella*, *Pseudomonas* and *E. coli*, and in numerous infection models, including a *Salmonella* calf ileal loop model, establishing their safety and low cytotoxicity [4,9,32]. Our ongoing work could lead to new treatment options for antibiotic-resistant Gram-negative bacterial infections. We are not proposing that pathogenicity inhibitors such as the SA compounds (or their derivatives) replace antibiotics; instead, they provide an attractive alternative with reduced evolutionary pressure for the development of resistance.

Finally, and significantly in this era of climate change, because AdhE spirosome length is unimportant for efficient conversion of acetyl-CoA to ethanol, unfractionated AdhE could be developed as a biological catalyst for biofuel production.

## Data Availability

The codes used to analyse and plot the total internal reflection fluorescence microscopy data have been deposited at Zenodo [33,34]. Any additional information required to reanalyse the data reported in this paper is available from the lead contact upon request. Supplementary material is available online [35].
